# Supplementary material for: A set of multi-entry identification keys to African frugivorous flies (Diptera, Tephritidae)
Source: Zookeys. 2014 Jul 24;(428):97–108. doi: 10.3897/zookeys.428.7366 (PMC4143993; doi:10.3897/zookeys.428.7366)
Supplement: Supplementary material 4 — Key to Capparimyia [file zookeys-428-097-s004.zip › SF4_ZooKeys_key to Capparimyia/lucid_player/help/help/using_subsets_of_features_and_entities.htm]

Using subsets of features and entities


# Using subsets of features and entities

*Subsets* are groups of features or
entities pre-defined by the author of the key.

For example, feature subsets in a key to beetles may allow you to restrict
*Features Available* to display
only wing features and head features, or all simple and easily observed
features. Entity subsets may allow you to restrict the *Entities*
panels to display only the beetles in a particular taxonomic subgroup,
or only common species.

*Subsets* are particularly useful
for keeping *Features Available*
and the *Entities* panels free
of unnecessary items. For example, if you have a key to plants and your
specimen has no fruits, it is useful to remove all fruit features using
subsets. Functions such as *Best*
will be more useful if you restrict *Features
Available* to show only features that it will be possible to address.

To choose a subset (if the key�s builder has specified subsets in the
key), open the *Subsets* list by
clicking the *Subsets* button  or choosing *Show Subsets*�
from the *Key* menu.

Note that you may choose
more than one subset.

If you are using subsets and need to keep the same subsets when you
restart the key, make sure that the *Key*
menu item *Keep Subsets* on *Restart* is checked. If this item is
unchecked, subsets will be set back to show all features and entities
when the key is restarted.
